# *trans*-Di­bromido­tetra­kis­(5-methyl-1*H*-pyrazole-κ*N* ^2^)manganese(II)

**DOI:** 10.1107/S2414314624002372

**Published:** 2024-03-19

**Authors:** Manikumar Athan, Soundararajan Krishnan, Nagarajan Loganathan

**Affiliations:** a School of Chemistry, Bharathidasan University, Tiruchirappalli 620 024, Tamilnadu, India; b Department of Chemistry, Periyar Maniammai Institute of Science and Technology, Vallam-613403, Thanjavur, Tamil Nadu, India; c UGC-Faculty Recharge Programme, University Grant Commission, New Delhi,India; Purdue University, USA

**Keywords:** manganese, coordination compound, crystal structure, heteroleptic complex, herringbone pattern, polymorphism

## Abstract

The title compound, *trans*-di­bromido­tetra­kis­(5-methyl-1*H*-pyrazole-κ*N* ^2^)manganese(II), [MnBr_2_(C_4_H_6_N_2_)_4_] or [Mn(3-MePzH)_4_Br_2_] (**1**) crystallizes in the triclinic *P*  space group with the cell parameters *a* = 7.6288 (3), *b* = 8.7530 (4), *c* = 9.3794 (4) Å and α = 90.707 (4), β = 106.138 (4), γ = 114.285 (5)°, *V* = 542.62 (5) Å^3^, *T* = 120 K. The asymmetric unit contains only half the mol­ecule with the manganese atom is situated on a crystallographic inversion center. The 3-MePzH ligands are present in an *AABB* type manner with two methyl groups pointing up and the other two down. The supra­molecular architecture is characterized by several inter­molecular C—H⋯N, N—H⋯Br, and C—H⋯π inter­actions. Earlier, a polymorphic structure of [Mn(3-MePzH)_4_Br_2_] (**2**) with a similar geometry and also an *AABB* arrangement for the pyrazole ligands was described [Reedijk *et al.* (1971 ▸). *Inorg. Chem*. **10**, 2594–2599; *a* = 8.802 (6), *b* = 9.695 (5), *c* = 7.613 (8) Å and α = 105.12 (4), β = 114.98 (4), γ = 92.90 (3)°, *V* = 558.826 (5) Å^3^, *T* = 295 K]. A varying supra­molecular pattern was reported, with the structure of **1** featuring a herringbone type pattern while that of structure **2** shows a pillared network type of arrangement along the *a* axis. A nickel complex [Ni(3-MePzH)_4_Br_2_] isomorphic to **1** and the analogous chloro derivatives of Fe^II^, Co^II^ and Cu^II^ are also known.

## Abstract 2

The title compound was synthesized using Mn(CO)_5_Br. The manganese atom is situated on a crystallographic inversion center. The supra­molecular architecture is characterized by several inter­molecular C—H⋯N, N—H⋯Br and C—H⋯π inter­actions.

## Graphical abstract

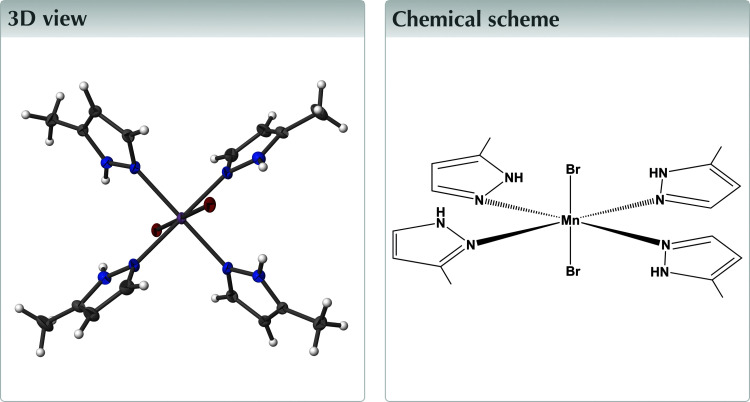

## Structure description

Earth-abundant transition metals such as manganese have received much attention owing to their numerous applications in biological, industrial, and material sciences (Constable *et al.*, 2021 ▸; Rice *et al.*, 2017 ▸; Zhang *et al.*, 2007 ▸; Dell, 2000 ▸). Apart from these applications, several mixed-valent multinuclear manganese cages have been assembled to understand their single mol­ecular magnetism (SMM) behavior (Zabala-Lekuona *et al.*, 2021 ▸). In addition, the famous Jacobson catalyst consisting of an Mn^II^–salen complex was developed for the enanti­oselective epoxidation of alkenes (Zhang *et al.*, 1990 ▸) while Mn^I^ carbonyls containing imidazolyl-based ligands have been used for the electrocatalytic-disproportionation of CO_2_ (Myren *et al.*, 2020 ▸). Likewise, many Mn^I^ carbonyls containing various *N*-heterocyclic ligands were developed as biomimicking models for hydrogenase enzymes (Xu *et al.*, 2016 ▸; Pan *et al.*, 2020 ▸.) and as CO-releasing mol­ecules (Mann, 2012 ▸; Cheng & Hu, 2021 ▸). Pyrazoles are one of the important classes of organic ligands used in many facets of coordination and organometallic chemistry (Trofimenko, 1972 ▸; Halcrow, 2009 ▸).

**Figure 1:**
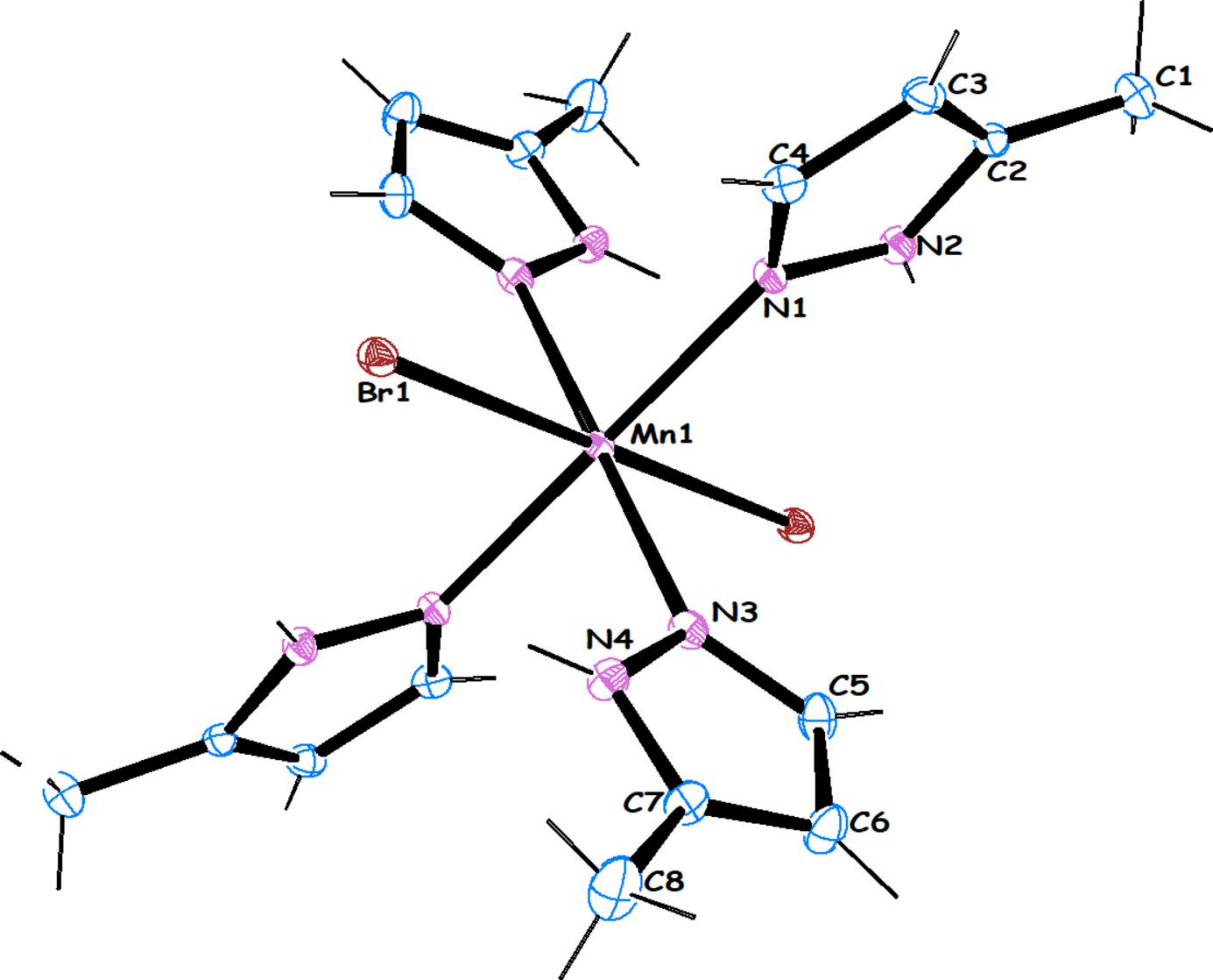
The mol­ecular structure of the title compound **1** with 50% probability displacement ellipsoids. The unlabeled atoms are related by symmetry.

We aim to synthesize various CO-releasing mol­ecules of the manganese family containing pyrazoles as primary ligands. In one such an attempt, a simple room-temperature stirring reaction involving the combination of Mn(CO)_5_Br, 5-methyl-1*H*-pyrazole and tri­ethyl­amine base (1:2:4) was found to release all CO mol­ecules and afforded yellow-colored crystals suitable for single-crystal X-ray diffraction analysis (SCXRD) from a di­chloro­methane-ethanol mixture (1:1) in qu­anti­tative yield. The SCXRD analysis reveals that it is *trans*-di­bromo tetra­kis­(5-methyl-1*H*-pyrazole-κ^2^ *N*)manganese(II) (**1**). In other words, Mn^I^ was oxidized *in situ* to Mn^II^ and an octa­hedral heteroleptic complex containing two bromo ligands *trans* to each other in the axial position and four neutral 5-methyl-1*H*-pyrazoles in the equatorial position was obtained (Fig. 1 ▸). The asymmetric unit contains half the mol­ecule with the manganese atom located on a crystallographic inversion center. The 3-MePzH ligands of the asymmetric unit are arranged in an *AABB* pattern with two neighboring pyrazole pointing upwards and the other two (their counterparts by inversion) downwards. The analysis reveals that it is a distorted octa­hedral complex with the axial distances to the larger bromine atoms [Mn1—Br1 = 2.7274 (3) Å] longer than the equatorial distances [Mn1—N1 = 2.251 (2) Å and Mn1—N3 = 2.261 (2) Å]. Angles at the manganese atom are close to 90° [N1—Mn1—Br1 = 89.10 (5)° and N3—Mn1—Br1 = 91.45 (5)°] and neighboring 3-MePzH rings are mutually perpendicular to each other with the dihedral angle between their planes being 87.08 (2)°.

**Figure 2:**
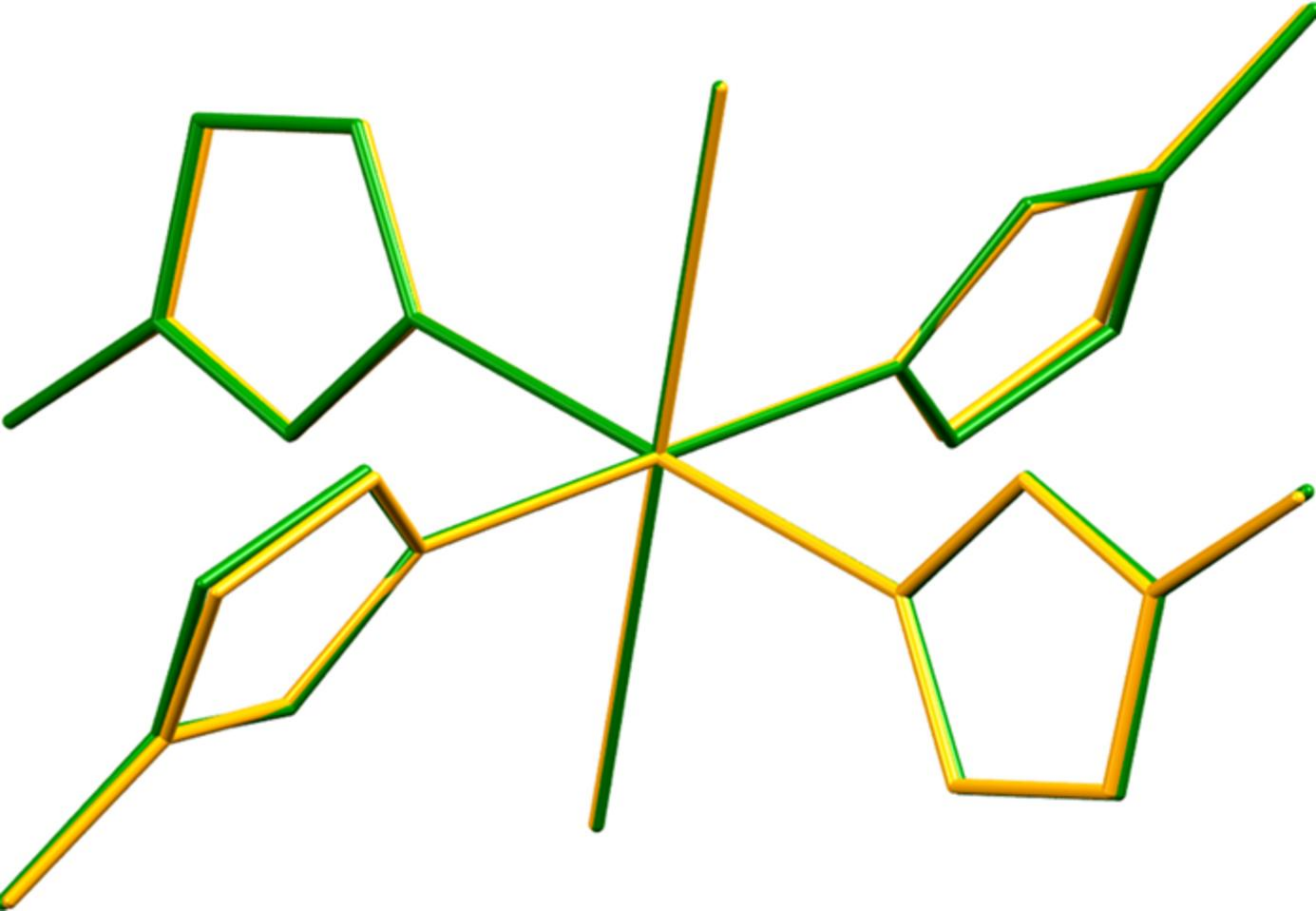
Overlay of the compounds **1** and **2** showing a slight deviation of 0.0612 Å (Mercury; Macrae *et al.*, 2020 ▸).

Earlier, many transition-metal pyrazoles were reported (Reedijk *et al.*, 1971 ▸; Bieller *et al.*, 2006 ▸; Cotton *et al.*, 2002 ▸; Nelana *et al.*, 2004 ▸; Khan *et al.*, 2014 ▸; Al Isawi *et al.*, 2023 ▸). In particular, Reedijk *et al.* (1971 ▸) synthesized many of the first transition-metal 5-methyl-1*H*-pyrazole complexes, including a polymorphic form of the title compound [Mn(3-MePzH)_4_Br_2_] (**2**), which was synthesized using MnBr_2_ and ethyl orthoformate as a dehydrating agent. It is inter­esting to note that the crystal data for compound **1** were collected at 120 K [*a* = 7.6288 (3), *b* = 8.7530 (4), *c* = 9.3794 (4) Å and α = 90.707 (4), β = 106.138 (4), γ = 114.285 (5)°, *V* = 542.62 (5) Å^3^] while compound **2** data were collected at 295 K [*a* = 8.802 (6), *b* = 9.695 (5), *c* = 7.613 (8) Å and α = 105.12 (4), β = 114.98 (4), γ = 92.90 (3)°, *V* = 558.826 (5) Å^3^]. A root-mean-square (r.m.s.) overlay of the mol­ecules of **1** and **2** using *Mercury 4.0* (Macrae *et al.*, 2020 ▸) is shown in Fig. 2 ▸ and reveals that in Reedijk’s polymorphic form, the 3-MePzH units are also placed in an *AABB* pattern with an r.m.s. deviation of 0.0612 Å. The analogous Mn^II^, Co^II^, Ni^II^, Cu^II^ bromo complexes isomorphic with Reedijk’s polymorph **2** were reported (Cotton *et al.*, 2002 ▸; Nelana *et al.*, 2004 ▸; Khan *et al.*, 2014 ▸) and the bond parameters of **1** and **2** are both in good agreement with those reported structures. Inter­estingly, the Ni^II^ bromo complex (Nelana *et al.*, 2004 ▸) is isomorphic with compound **1**. It was synthesized using (1,2-di­meth­oxy­ethane)_2_NiBr_2_ as the metal source.

**Figure 3:**
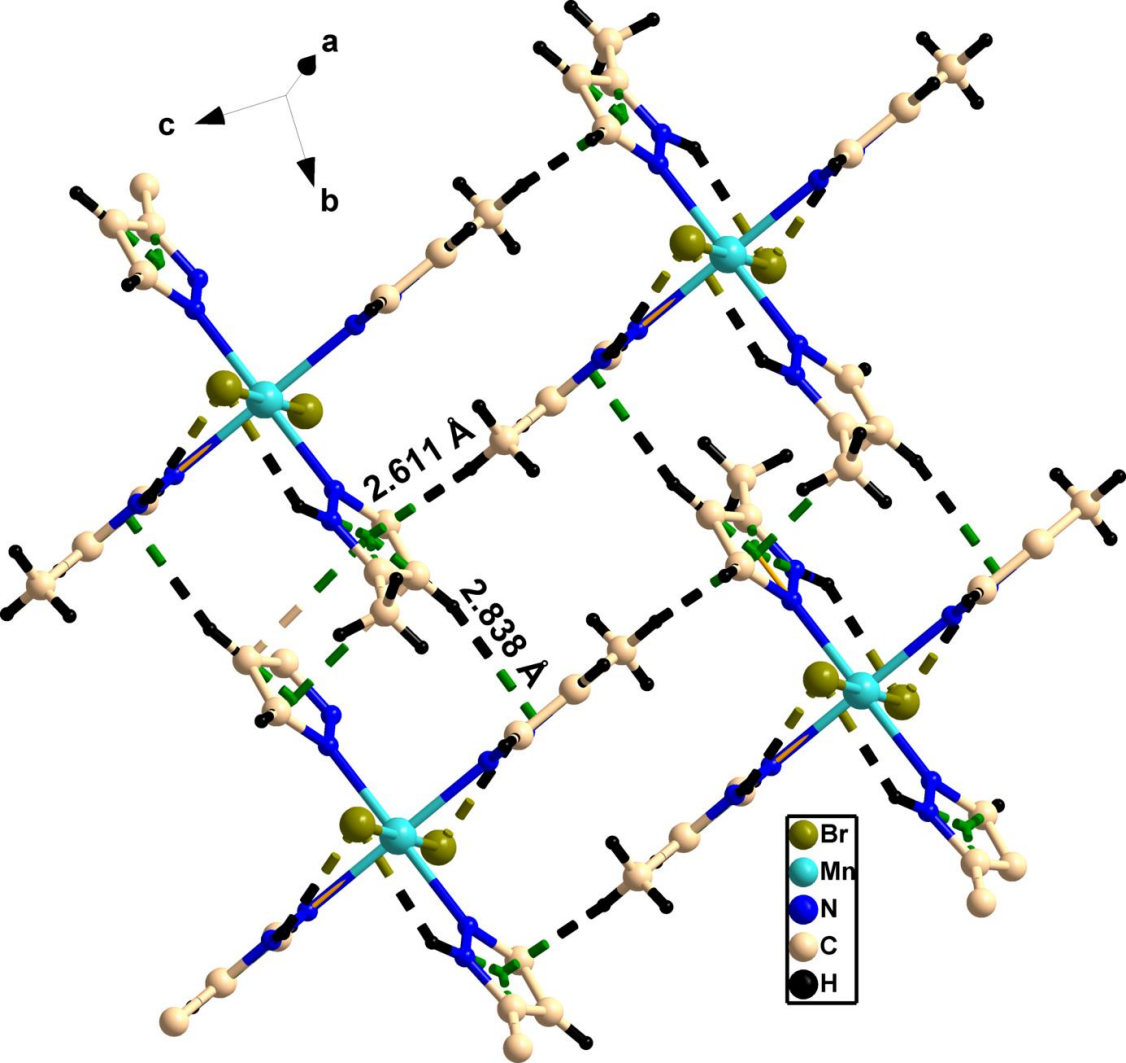
Perspective view of the supra­molecular pattern of compound **1** showing the presence of inter­molecular C—H⋯π inter­actions.

**Figure 4:**
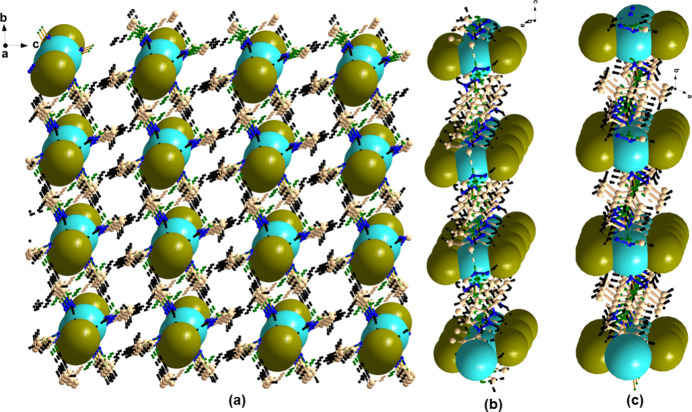
Supra­molecular architecture of compound **1**. (*a*) View along the *a* axis showing a herringbone-type arrangement; (*b*) and (*c*) the stacking of Br—Mn—Br along the respective axes.

**Figure 5:**
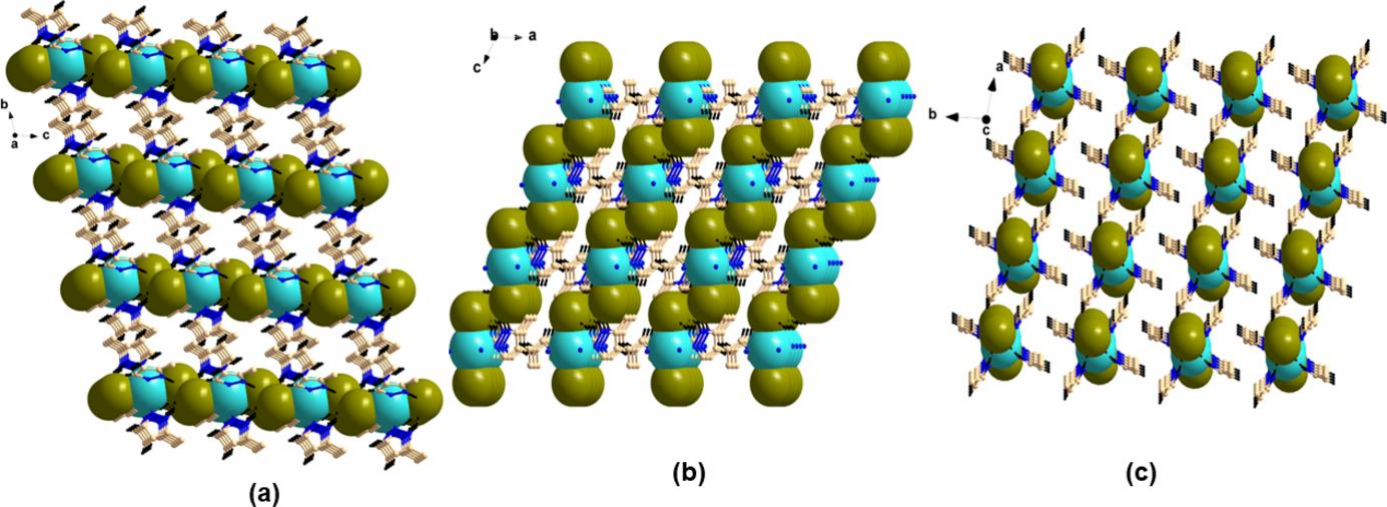
Supra­molecular architecture of compound **2**. (*a*) View along the *a* axis showing a pillared network arrangement; (*b*) a view along the *b* axis showing the zigzag pattern of Br—Mn—Br and (*c*) a view along the *c* axis.

Compound **1** contains various intra- and inter­molecular inter­actions in the form of N—H⋯Br and C—H⋯N inter­actions as well as C—H⋯π inter­actions (see Table 1 ▸). A perspective view of the supra­molecular architecture of **1** is given in Fig. 3 ▸, which shows the presence of the various C—H⋯π inter­actions, leading to the formation of a herringbone-type of arrangement (Fig. 4 ▸ *a*) along the *a* axis. In contrast, a pillared network along the *a* axis is seen in the structure of **2** (Fig. 5 ▸ *a*). Further investigation reveals that along the *b* axis, the Br—Mn—Br moieties are stacked one over another in compound **1** while in **2**, they are arranged in a zigzag fashion (Fig. 4 ▸ *b* and 5*b*). The view along *c* axis is also different in both the compounds (Fig. 4 ▸ *c* and 5*c*). Overall, the supra­molecular architectures clearly distinguish the two polymorphic forms **1** and **2**.

## Synthesis and crystallization

50 mg (0.19 mmol) of Mn(CO)_5_Br [bromo­penta­carbonyl­manganese(I)] and 30.6 µ*L* (0.38 mmol) of 5-methyl-1*H*-pyrazole were dissolved in 20 ml of ethanol. After stirring for a few minutes, 105 µ*L* (0.76 mmol) of tri­ethyl­amine were added to the reaction mixture and the resultant straw-yellow-colored solution was stirred at room temperature for 20 h. Light-yellow crystals were obtained by the slow evaporation method of a 1:1 di­chloro­methane–ethanol solvent mixture. Crystal yield 60%. ESI–MS data: *m*/*z* 540.53290 [*M* – H]^+^.

## Refinement

Crystal data, data collection and structure refinement details are summarized in Table 2 ▸.

## Supplementary Material

Crystal structure: contains datablock(s) I. DOI: 10.1107/S2414314624002372/zl4064sup1.cif

Structure factors: contains datablock(s) I. DOI: 10.1107/S2414314624002372/zl4064Isup2.hkl

CCDC reference: 2322181

Additional supporting information:  crystallographic information; 3D view; checkCIF report

## Figures and Tables

**Table 1:** Hydrogen-bond geometry (Å, °) *Cg*1 and *Cg*2 are the centroids of the N1/N2/C2–C4 and N3/N4/ C5–C7 rings, respectively.

*D*—H⋯*A*	*D*—H	H⋯*A*	*D*⋯*A*	*D*—H⋯*A*
N2—H2⋯Br1	0.84 (3)	2.70 (3)	3.343 (2)	134 (2)
C1—H1*C*⋯Br1^i^	0.98	3.11	3.951 (3)	145
N2—H2⋯Br1	0.84 (3)	2.70 (3)	3.343 (2)	134 (2)
N2—H2⋯Br1^i^	0.84 (3)	3.05 (3)	3.704 (2)	137 (2)
N4—H7⋯Br1^ii^	0.80 (3)	3.00 (3)	3.636 (2)	138 (3)
N4—H7⋯Br1^iii^	0.80 (3)	2.76 (3)	3.351 (2)	132 (3)
C3—H3⋯N4^iv^	0.95	2.76	3.659 (3)	158
C8—H8*A*⋯N2^v^	0.98	2.86	3.744 (3)	151
C8—H8*A*⋯*Cg*1^v^	0.98	2.61	3.586 (3)	173
C3—H3⋯*Cg*2^vi^	0.95	2.84	3.667 (4)	146

Symmetry codes: (i) 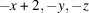 ; (ii) 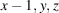 ; (iii) 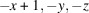 ; (iv) 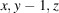 ; (v) 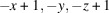 ; (vi) 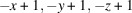 .

**Table 2:** Experimental details

Crystal data	
Chemical formula	[MnBr_2_(C_4_H_6_N_2_)_4_]
*M*_r_	543.19
Crystal system, space group	Triclinic, *P* 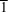
Temperature (K)	120
*a*, *b*, *c* (Å)	7.6288 (3), 8.7530 (4), 9.3794 (4)
α, β, γ (°)	90.707 (4), 106.138 (4), 114.285 (5)
*V* (Å^3^)	542.62 (5)
*Z*	1
Radiation type	Mo *K*α
μ (mm^−1^)	4.31
Crystal size (mm)	0.17 × 0.14 × 0.12

Data collection	
Diffractometer	XtaLAB AFC12 (RINC): Kappa single
Absorption correction	Multi-scan (*CrysAlis PRO*; Rigaku OD, 2017 ▸)
*T*_min_, *T*_max_	0.706, 1.000
No. of measured, independent and observed [*I* > 2σ(*I*)] reflections	11690, 2574, 2115
*R*_int_	0.060
(sin θ/λ)_max_ (Å^−1^)	0.680

Refinement	
*R*[*F* ^2^ > 2σ(*F* ^2^)], *wR*(*F* ^2^), *S*	0.032, 0.064, 1.04
No. of reflections	2574
No. of parameters	134
H-atom treatment	H atoms treated by a mixture of independent and constrained refinement
Δρ_max_, Δρ_min_ (e Å^−3^)	0.50, −0.38

Computer programs: *CrysAlis PRO* (Rigaku OD, 2017 ▸), *SHELXT* (Sheldrick, 2015*a* ▸), *SHELXL2019/3* (Sheldrick, 2015*b* ▸), *ORTEP-3 for Windows2020* (Farrugia, 2012 ▸), Diamond (Brandenburg *et al.*, 2014 ▸) and *publCIF* (Westrip, 2010 ▸).

## full crystallographic data

### Crystal data

**Table:**

[MnBr_2_(C_4_H_6_N_2_)_4_]	*Z* = 1
*M**_r_* = 543.19	*F*(000) = 271
Triclinic, *P*1	*D*_x_ = 1.662 Mg m^−^^3^
*a* = 7.6288 (3) Å	Mo *K*α radiation, λ = 0.71073 Å
*b* = 8.7530 (4) Å	Cell parameters from 4000 reflections
*c* = 9.3794 (4) Å	θ = 3.1–28.0°
α = 90.707 (4)°	µ = 4.31 mm^−^^1^
β = 106.138 (4)°	*T* = 120 K
γ = 114.285 (5)°	Block, yellow
*V* = 542.62 (5) Å^3^	0.17 × 0.14 × 0.12 mm

### Data collection

**Table:**

XtaLAB AFC12 (RINC): Kappa single diffractometer	*R*_int_ = 0.060
Radiation source: fine-focus sealed X-ray tube	θ_max_ = 28.9°, θ_min_ = 3.1°
ω scans	*h* = −10→10
Absorption correction: multi-scan (CrysAlisPro; Rigaku OD, 2017)	*k* = −10→11
*T*_min_ = 0.706, *T*_max_ = 1.000	*l* = −12→11
11690 measured reflections	3 standard reflections every 20 reflections
2574 independent reflections	intensity decay: none
2115 reflections with *I* > 2σ(*I*)	

### Refinement

**Table:**

Refinement on *F*^2^	Primary atom site location: dual
Least-squares matrix: full	Secondary atom site location: difference Fourier map
*R*[*F*^2^ > 2σ(*F*^2^)] = 0.032	Hydrogen site location: mixed
*wR*(*F*^2^) = 0.064	H atoms treated by a mixture of independent and constrained refinement
*S* = 1.04	*w* = 1/[σ^2^(*F*_o_^2^) + (0.0225*P*)^2^ + 0.1264*P*] where *P* = (*F*_o_^2^ + 2*F*_c_^2^)/3
2574 reflections	(Δ/σ)_max_ < 0.001
134 parameters	Δρ_max_ = 0.50 e Å^−^^3^
0 restraints	Δρ_min_ = −0.37 e Å^−^^3^

### Special details

**Table:**

Geometry. All esds (except the esd in the dihedral angle between two l.s. planes) are estimated using the full covariance matrix. The cell esds are taken into account individually in the estimation of esds in distances, angles and torsion angles; correlations between esds in cell parameters are only used when they are defined by crystal symmetry. An approximate (isotropic) treatment of cell esds is used for estimating esds involving l.s. planes.

### Fractional atomic coordinates and isotropic or equivalent isotropic displacement parameters (Å^2^)

**Table:**

	*x*	*y*	*z*	*U*_iso_*/*U*_eq_	
C1	0.8781 (4)	−0.4193 (4)	0.2379 (3)	0.0248 (6)	
H1A	0.841583	−0.535204	0.195589	0.037*	
H1B	0.924272	−0.407646	0.347700	0.037*	
H1C	0.986772	−0.339348	0.203390	0.037*	
C2	0.6971 (4)	−0.3823 (3)	0.1878 (3)	0.0177 (5)	
C3	0.5014 (4)	−0.4665 (3)	0.1909 (3)	0.0196 (6)	
H3	0.443446	−0.571543	0.225950	0.024*	
C4	0.4061 (4)	−0.3654 (3)	0.1319 (3)	0.0191 (5)	
H4	0.268506	−0.392289	0.120452	0.023*	
C5	0.6662 (4)	0.1767 (4)	0.3552 (3)	0.0234 (6)	
H5	0.803196	0.199834	0.368271	0.028*	
C6	0.5970 (4)	0.2174 (4)	0.4670 (3)	0.0251 (6)	
H6	0.675254	0.271808	0.566727	0.030*	
C7	0.3930 (4)	0.1625 (3)	0.4026 (3)	0.0201 (6)	
C8	0.2336 (4)	0.1681 (4)	0.4618 (3)	0.0329 (7)	
H8A	0.264306	0.152549	0.567683	0.049*	
H8B	0.101977	0.077534	0.404452	0.049*	
H8C	0.229412	0.278049	0.452166	0.049*	
Br1	0.91038 (4)	0.16217 (3)	0.06734 (3)	0.01922 (10)	
Mn1	0.500000	0.000000	0.000000	0.01496 (13)	
N1	0.5313 (3)	−0.2264 (3)	0.0935 (2)	0.0169 (4)	
N2	0.7090 (3)	−0.2395 (3)	0.1301 (2)	0.0177 (5)	
N3	0.5156 (3)	0.1016 (3)	0.2283 (2)	0.0175 (5)	
N4	0.3507 (3)	0.0941 (3)	0.2610 (2)	0.0193 (5)	
H2	0.804 (4)	−0.166 (4)	0.106 (3)	0.013 (7)*	
H7	0.244 (5)	0.058 (4)	0.196 (3)	0.025 (8)*	

### Atomic displacement parameters (Å^2^)

**Table:**

	*U*^11^	*U*^22^	*U*^33^	*U*^12^	*U*^13^	*U*^23^
C1	0.0248 (15)	0.0259 (15)	0.0293 (15)	0.0154 (13)	0.0098 (12)	0.0085 (12)
C2	0.0219 (13)	0.0169 (13)	0.0145 (12)	0.0100 (11)	0.0038 (10)	0.0005 (10)
C3	0.0258 (14)	0.0163 (13)	0.0177 (13)	0.0092 (11)	0.0079 (11)	0.0036 (10)
C4	0.0184 (13)	0.0187 (14)	0.0208 (13)	0.0069 (11)	0.0088 (10)	0.0018 (10)
C5	0.0165 (13)	0.0293 (16)	0.0253 (14)	0.0116 (12)	0.0052 (11)	0.0054 (12)
C6	0.0237 (14)	0.0349 (17)	0.0161 (13)	0.0124 (13)	0.0058 (11)	0.0008 (11)
C7	0.0216 (14)	0.0229 (14)	0.0165 (13)	0.0086 (11)	0.0087 (11)	0.0027 (10)
C8	0.0276 (16)	0.055 (2)	0.0204 (14)	0.0201 (15)	0.0104 (12)	0.0017 (13)
Br1	0.01626 (14)	0.01839 (15)	0.02480 (15)	0.00854 (11)	0.00746 (10)	0.00161 (10)
Mn1	0.0161 (3)	0.0151 (3)	0.0174 (3)	0.0090 (2)	0.0070 (2)	0.0023 (2)
N1	0.0180 (11)	0.0168 (11)	0.0181 (11)	0.0106 (9)	0.0042 (9)	0.0020 (8)
N2	0.0148 (11)	0.0175 (12)	0.0208 (12)	0.0061 (10)	0.0065 (9)	0.0040 (9)
N3	0.0163 (11)	0.0204 (12)	0.0203 (11)	0.0098 (9)	0.0092 (9)	0.0030 (9)
N4	0.0159 (12)	0.0201 (12)	0.0195 (12)	0.0054 (10)	0.0056 (10)	0.0013 (9)

### Geometric parameters (Å, º)

**Table:**

C1—C2	1.497 (3)	C7—N4	1.346 (3)
C1—H1A	0.9800	C7—C8	1.488 (4)
C1—H1B	0.9800	C8—H8A	0.9800
C1—H1C	0.9800	C8—H8B	0.9800
C2—N2	1.348 (3)	C8—H8C	0.9800
C2—C3	1.376 (4)	Br1—Br1	0.0000 (10)
C3—C4	1.391 (4)	Br1—Mn1	2.7274 (3)
C3—H3	0.9500	Mn1—N1	2.251 (2)
C4—N1	1.330 (3)	Mn1—N1^i^	2.251 (2)
C4—H4	0.9500	Mn1—N3	2.2612 (19)
C5—N3	1.330 (3)	Mn1—N3^i^	2.2613 (19)
C5—C6	1.400 (4)	N1—N2	1.356 (3)
C5—H5	0.9500	N2—H2	0.84 (3)
C6—C7	1.368 (4)	N3—N4	1.352 (3)
C6—H6	0.9500	N4—H7	0.80 (3)
			
C2—C1—H1A	109.5	N1—Mn1—N3	89.74 (7)
C2—C1—H1B	109.5	N1^i^—Mn1—N3	90.26 (7)
H1A—C1—H1B	109.5	N1—Mn1—N3^i^	90.26 (7)
C2—C1—H1C	109.5	N1^i^—Mn1—N3^i^	89.74 (7)
H1A—C1—H1C	109.5	N3—Mn1—N3^i^	180.0
H1B—C1—H1C	109.5	N1—Mn1—Br1	89.10 (5)
N2—C2—C3	105.9 (2)	N1^i^—Mn1—Br1	90.90 (5)
N2—C2—C1	121.2 (2)	N3—Mn1—Br1	91.45 (5)
C3—C2—C1	132.9 (2)	N3^i^—Mn1—Br1	88.55 (5)
C2—C3—C4	105.5 (2)	N1—Mn1—Br1	89.10 (5)
C2—C3—H3	127.3	N1^i^—Mn1—Br1	90.90 (5)
C4—C3—H3	127.3	N3—Mn1—Br1	91.45 (5)
N1—C4—C3	111.7 (2)	N3^i^—Mn1—Br1	88.55 (5)
N1—C4—H4	124.1	Br1—Mn1—Br1	0.000 (16)
C3—C4—H4	124.1	N1—Mn1—Br1^i^	90.90 (5)
N3—C5—C6	111.3 (2)	N1^i^—Mn1—Br1^i^	89.10 (5)
N3—C5—H5	124.3	N3—Mn1—Br1^i^	88.55 (5)
C6—C5—H5	124.3	N3^i^—Mn1—Br1^i^	91.45 (5)
C7—C6—C5	105.6 (2)	Br1—Mn1—Br1^i^	180.0
C7—C6—H6	127.2	Br1—Mn1—Br1^i^	180.0
C5—C6—H6	127.2	C4—N1—N2	104.0 (2)
N4—C7—C6	105.9 (2)	C4—N1—Mn1	133.75 (17)
N4—C7—C8	122.0 (2)	N2—N1—Mn1	122.16 (16)
C6—C7—C8	132.1 (2)	C2—N2—N1	112.9 (2)
C7—C8—H8A	109.5	C2—N2—H2	129.5 (18)
C7—C8—H8B	109.5	N1—N2—H2	117.2 (18)
H8A—C8—H8B	109.5	C5—N3—N4	104.0 (2)
C7—C8—H8C	109.5	C5—N3—Mn1	133.35 (17)
H8A—C8—H8C	109.5	N4—N3—Mn1	122.65 (15)
H8B—C8—H8C	109.5	C7—N4—N3	113.2 (2)
Br1—Br1—Mn1	0.00 (2)	C7—N4—H7	127 (2)
N1—Mn1—N1^i^	180.0	N3—N4—H7	119 (2)
			
N2—C2—C3—C4	0.4 (3)	C1—C2—N2—N1	−178.9 (2)
C1—C2—C3—C4	178.2 (3)	C4—N1—N2—C2	0.8 (3)
C2—C3—C4—N1	0.1 (3)	Mn1—N1—N2—C2	178.32 (15)
N3—C5—C6—C7	0.3 (3)	C6—C5—N3—N4	−0.4 (3)
C5—C6—C7—N4	0.0 (3)	C6—C5—N3—Mn1	179.66 (18)
C5—C6—C7—C8	−180.0 (3)	C6—C7—N4—N3	−0.3 (3)
C3—C4—N1—N2	−0.5 (3)	C8—C7—N4—N3	179.7 (2)
C3—C4—N1—Mn1	−177.60 (16)	C5—N3—N4—C7	0.4 (3)
C3—C2—N2—N1	−0.8 (3)	Mn1—N3—N4—C7	−179.64 (17)

Symmetry code: (i) −*x*+1, −*y*, −*z*.

### Hydrogen-bond geometry (Å, º)

*Cg*1 and *Cg*2 are the centroids of the N1/N2/C2–C4 and N3/N4/ C5–C7 rings, respectively.

**Table:**

*D*—H···*A*	*D*—H	H···*A*	*D*···*A*	*D*—H···*A*
N2—H2···Br1	0.84 (3)	2.70 (3)	3.343 (2)	134 (2)
C1—H1*C*···Br1^ii^	0.98	3.11	3.951 (3)	145
N2—H2···Br1	0.84 (3)	2.70 (3)	3.343 (2)	134 (2)
N2—H2···Br1^ii^	0.84 (3)	3.05 (3)	3.704 (2)	137 (2)
N4—H7···Br1^iii^	0.80 (3)	3.00 (3)	3.636 (2)	138 (3)
N4—H7···Br1^i^	0.80 (3)	2.76 (3)	3.351 (2)	132 (3)
C3—H3···N4^iv^	0.95	2.76	3.659 (3)	158
C8—H8*A*···N2^v^	0.98	2.86	3.744 (3)	151
C8—H8*A*···*Cg*1^v^	0.98	2.61	3.586 (3)	173
C3—H3···*Cg*2^vi^	0.95	2.84	3.667 (4)	146

Symmetry codes: (i) −*x*+1, −*y*, −*z*; (ii) −*x*+2, −*y*, −*z*; (iii) *x*−1, *y*, *z*; (iv) *x*, *y*−1, *z*; (v) −*x*+1, −*y*, −*z*+1; (vi) −*x*+1, −*y*+1, −*z*+1.
